# Supplementary material for: Silage Fermentation: A Potential Biological Approach for the Long-Term Preservation and Recycling of Polyphenols and Terpenes in Globe Artichoke (Cynara scolymus L.) By-Products
Source: Molecules. 2020 Jul 21;25(14):3302. doi: 10.3390/molecules25143302 (PMC7397312; doi:10.3390/molecules25143302)
Supplement: Supplementary file 1 [file molecules-25-03302-s001.pdf]

Supplementary material

Silage Fermentation: A Potential Biological Approach for the Long-term Preservation and Recycling of Polyphenols and Terpenes in Globe Artichoke (*Cynara scolymus* L.) By-products

Zhuoyan Fan[1], Kai Chen[1], Lingyin Ban[1], Yu Mao[1], Caiyun Hou[1]*,
Jingming Li[1]*

[1]*College of Food Science and Nutritional Engineering,
China Agricultural University, Beijing, China*

[1]* Correspondence authors: lijingming@cau.edu.cn

Tel: +86-010-62346869;

[1]* Correspondence authors: cyhou@cau.edu.cn

Table SM 1: The limit of detection (LOD) and limit of quantification (LOQ) and linear equation of polyphenols

Standard	Standard curve	R^2	Linear range （μg/L）	LOD （μg/L）	LOQ （μg/L）
Salicylic acid	y=293.255x-287.844	0.9994	10-500	1.68	5.60
Caffeic acid	y=52.8777x+2122.5	0.9997	58.5-11700	4.26	14.21
Vanillic acid	y=2.81622x-29.6034	0.9997	50-1000	14.01	46.70
Ferulic acid	y=10.5697x-86.114	0.9993	20-1000	5.65	18.83
Luteolin	y=3.18188x+1874.48	0.9993	500-10000	10.22	34.07
Chlorogenic acid	y=20.4979x+732.94	0.9999	200-10000	12.94	43.13
Syringic acid	y=4.74307x-37.5689	0.9993	21.6-2160	6.09	20.30
Cynarin	y=8.50171x+42.9748	0.9997	20-10000	6.40	21.34
Gallic acid	y=13.865x-197.859	0.9991	50-5000	14.41	48.03
Apigenin	y=0.048618x-0.904486	0.9997	500-10000	2.92	9.74
Protocatechuic acid	y=68.4094x-338.16	0.9993	50-2500	4.12	13.73
p-Coumaric acid	y=93.4543x-63.5635	0.9992	50-2500	3.72	12.40
Phlorizin	y=26.1328x-586.255	0.9995	50-2500	2.96	9.87
p-Hydroxybenzoic acid	y=95.7398x-179.144	0.9998	20-1000	3.71	12.38
(-)-Epicatechin gallate	y=8.08908x+0.641407	0.9998	20-500	4.96	16.54

Table SM 2 : Characteristic ions of polyphenols

Compounds	$[M-H]^-$ m/z	HPLC-ESI(−)-MS^2 expt m/z (% base peak)	Reference
1-*O*-caffeoylquinic acid	353	MS^2 [353]: 191 (100), 197 (5)	
4-*O*-caffeoylquinic acid	353	MS2 [353]: 173 (100), 179 (53), 191 (16), 135 (12)	[1]
Apigenin 7-*O*-glucuronide	445	MS2 [445]: 269 (100), 175 (20)	

Table SM 3: Electron Impact Mass Spectra (EI-MS) of the sesquiterpene lactones and the pentacyclic triterpenes identified in the form of TMS derivatives

Compounds	Fragment ions of EI-MS (70 eV), m/z	Reference
Ψ-Taraxasterol-TMS derivative	498$[M]^+$ (6), 483(2), 408(12), 218(10), 203(16), 189(100), 175(19)	[2]
Taraxasterol-TMS derivative	498$[M]^+$ (8), 483(3), 408(8), 218(9), 203(21), 189(100), 175(21)	

Table SM 4: Identification of terpenoids in the fat soluble extract of artichoke

RT /min	Compound	RI	RI_{ref}	Qualitative methods
	**Pentacyclic triterpenes**			
51.024	*α*-Amyrin	3385.0	3379.3	A/B
51.293	Lupeol	3396.1	3390.8	A/B
53.325	*Ψ*-Taraxasterol	3480.5	-	C [2,3]
53.623	Taraxasterol	3493.2	-	C [2,3]
	**Sterols**			
48.775	Stigmasterol	3290.3	3285.6	A/B
50.188	*β*-Sitosterol	3349.1	3344	A/B

RT: retention time; RI: retention index; RI_{ref}: retention index in reference; A: Qualitative by National Institute of Standard and Technology (NIST) mass spectral library; B:Qualitative by standard sample; C: Qualitative analysis by mass spectrometry information in reference.

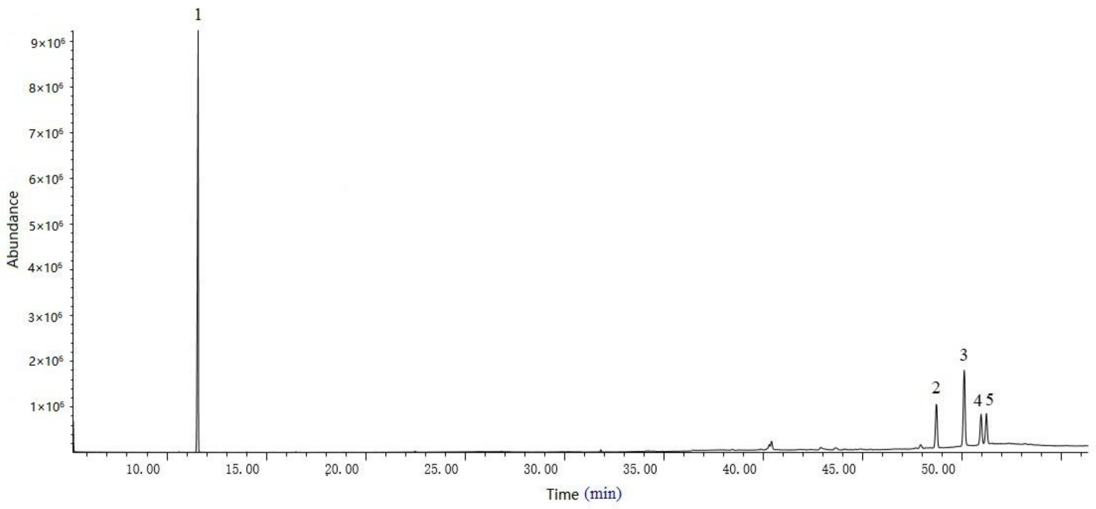

Fig SM 1. Total ion efflux of terpene standards. 1. N-hexadecane; 2. Stigmasterol; 3.*β*- sitosterol; 4.*α*-Amyrin; 5. Lupeol.

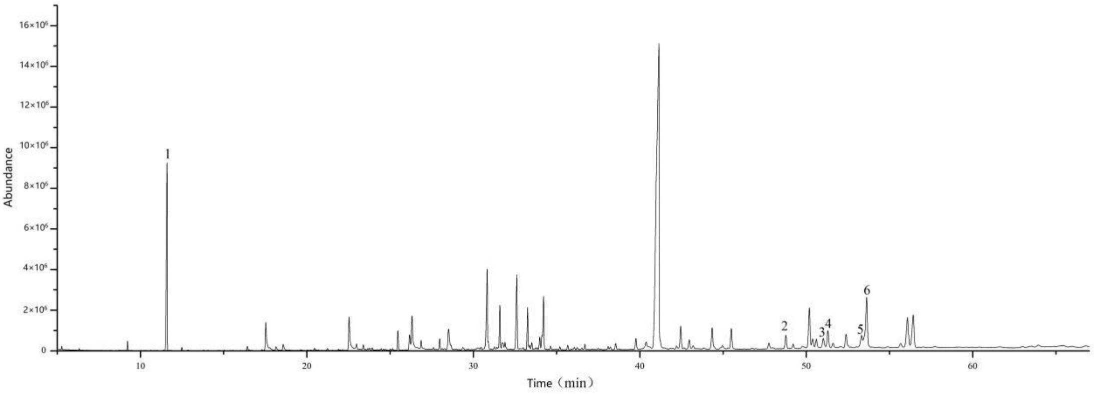

Fig SM 2. Total ion outflow of terpenoids from artichoke by-product. 1. N-hexadecane; 2. Stigmasterol; 3.*β*- sitosterol; 3.*α*-Amyrin; 4. Lupeol; 5.*Ψ*-Taraxasterol; 6. Taraxasterol

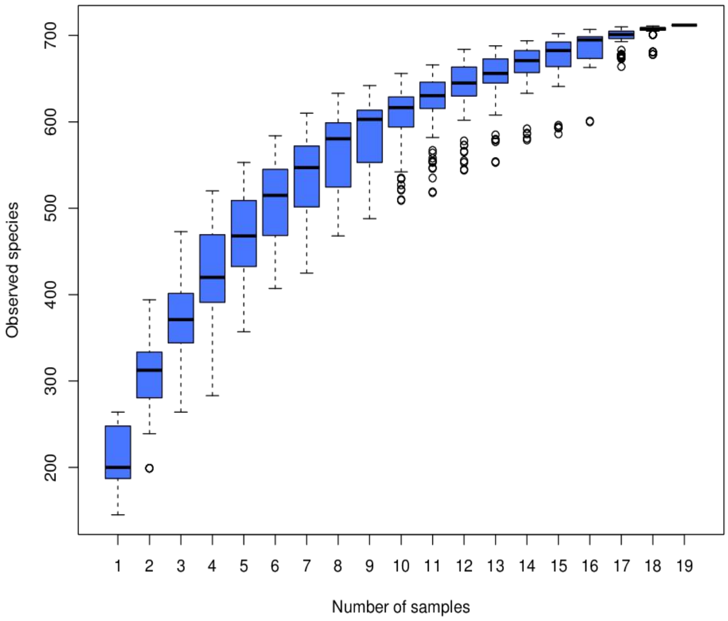

Fig SM 3. Species accumulation boxplot.

References:

1. Schütz, K., Kammerer, D., Carle, R., Schieber, A., Identification and Quantification of Caffeoylquinic Acids and Flavonoids from Artichoke (*Cynara scolymus L.*) Heads, Juice, and Pomace by HPLC-DAD-ESI/MS n. *J. Agr. Food Chem*. **2004**, *52*, 4090-4096.

2. Ramos, P.A.B., Guerra, N.R., Guerreiro, O., Freire, C. S. R., Silva, A. M. S., Duarte, M. F., Silvestre, A. J. D., Lipophilic Extracts of *Cynara cardunculus L.* var. altilis (DC): A Source of Valuable Bioactive Terpenic Compounds. *J. Agr. Food Chem*. **2013**, *61*, 8420-8429.

3. Wang, Z., Guhling, O., Yao, R., Li, F., Yeats, T.H., Rose, J.K. C., Jetter, R., Two Oxidosqualene Cyclases Responsible for Biosynthesis of Tomato Fruit Cuticular Triterpenoids. *Plant Physiol.* **2011**, *155*, 540-552.
